# Threshold detection by fitting segmented regression models in Microsoft Excel

**DOI:** 10.1016/j.mex.2025.103573

**Published:** 2025-08-19

**Authors:** Amy J Hopper, Angus M Brown

**Affiliations:** a School of Life Sciences, University of Nottingham, Nottingham NG7 2UH, UK; b Department of Neurology, University of Washington, Seattle WA 98195, USA

**Keywords:** Microsoft Excel, Solver, Least squares, Regression

## Abstract

We present a generally applicable method for segmented regression analysis, which is suitable for describing data that follow two distinct functions that meet at an unknown transition or break point and is particularly useful for detecting thresholds. Although segmented regression analysis is available in Matlab and R, it requires specialist knowledge beyond the expertise of many researchers. We illustrate a method for fitting experimental data with two distinct segmented linear functions using SOLVER, freely available with Microsoft Excel. A spreadsheet template is created for input of experimental data and the fit between the model and the data optimised using SOLVER’s iterative least squares fitting routine to estimate the transition point. We then demonstrate how the method can be expanded to incorporate combinations of linear and non-linear functions. The method is ideal for rapid processing of data and sufficiently flexible to allow for modifications to functions when required.

•Experimental data that follow a model comprising two distinct functions that meet at an unknown transition point is amenable to segmented regression analysis.

•We describe a method that uses SOLVER, an add-in that is freely available with Microsoft Excel, to carry out this analysis. •The method does not require any specialist programming knowledge.

## Graphical abstract

**Figure:**
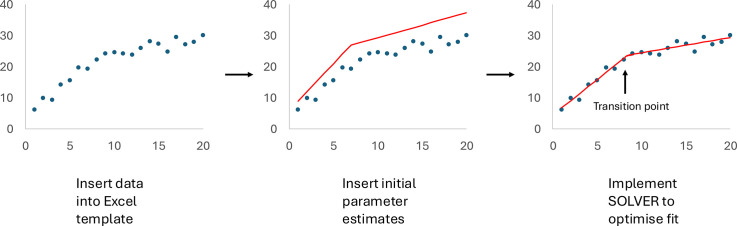

## Specifications table

**Table:** 

**Subject area**	Neuroscience
**More specific subject area**	Threshold detection
**Name of your method**	Segmented regression analysis
**Name and reference of original method**	How to use Excel in Analytical Chemistry and in General Scientific Data Analysis by Robert de Levie. Cambridge. Chapter 3. 2001
**Resource availability**	A spreadsheet template is available on request from the corresponding author

## Background

Scientific data are routinely described by models where the dependent variable is continuously related to the independent variable according to *y* = *f*(x), where *f* may be a simple linear function or a more complex non-linear function. However, some data follow two different functions on opposite sides of an unknown transition point and are more realistically described by a model comprising a sequence of two functions, which meet at the transition point. These data are amenable to segmented regression analysis. There are numerous examples of such biological relationships [1], but of particular interest are systems where, once a threshold has been reached, the function abruptly changes. The appeal of fitting such data is obvious since it allows quantification of the thresholds.

Initial attempts to meaningfully address segmented regression analysis commenced in 1960 [2], with further progress in the following decades [[3], [4], [5], [6]]. The complex calculations underlying the apparently simple process of estimating transition points ensured that such analysis remained the exclusive province of the mathematically expert. Consequently, segmented regression analysis is absent from standard curve fitting packages such as GraphPad Prism. The introduction of programmes such as MATLAB [7] and R [8] expanded access to segmented regression analysis, but these programmes rely on specialist knowledge of bespoke functions, and cannot be recommended for non-experts.

The purpose of this present paper is to introduce a simple, generally applicable method for segmented regression analysis. The method uses the SOLVER add in, freely available with the ubiquitous spreadsheet Microsoft Excel, to create a template that uses an iterative least squares fitting routine to estimate the transition point. We demonstrate the conventional linear-linear model with example data, then expand the method to include combinations of linear and non-linaer functions.

## Method details

### Configuring the spreadsheet

We recommend readers familiarise themselves with installation and application of the SOLVER add in [9,10] and its use in non-linear regression analysis [[11], [12], [13]] before proceeding to segmented analysis. The steps to create the spreadsheet template for carrying out segmented regression analysis comprising two linear functions are as follows.

1. The sample data, expressed as (x, y) coordinates, are entered onto the spreadsheet in two adjacent columns (A & B). Column A is headed x and Column B is headed y_data_ (Table 1).

**Table 1:** Spreadsheet template for segmented regression analysis. *Ad hoc* data that can be fit by a model comprising two linear functions, which meet at a transition point, are illustrated. Data are entered onto the spreadsheet, where Column A contains the independent variable, Column B contains the observed dependent data (y_data_) and Column C contains the model (y_model_). Initial parameter estimates are entered in cells F2 to F5. In-cell formulae in Column C use the parameter estimates to calculate the preliminary fit, which in turn is used to calculate SS_res_ and R^2^.

	A	B	C	D	E	F
**1**	**x**	**y_data_**	**y_model_**			
**2**	1	6.4	=IF(A2<cc,ao+ai*A2,ao+cc*(ai-bi)+bi*A2)		**a_o_**	6.00
**3**	2	10.1	=IF(A3<cc,ao+ai*A3,ao+cc*(ai-bi)+bi*A3)		**a_i_**	3.00
**4**	3	9.5	=IF(A4<cc,ao+ai*A4,ao+cc*(ai-bi)+bi*A4)		**b_i_**	0.80
**5**	4	14.3	=IF(A5<cc,ao+ai*A5,ao+cc*(ai-bi)+bi*A5)		**cc**	7.00
**6**	5	15.8	=IF(A6<cc,ao+ai*A6,ao+cc*(ai-bi)+bi*A6)		**y_mean_**	=AVERAGE(B2:B21)
**7**	6	19.8	=IF(A7<cc,ao+ai*A7,ao+cc*(ai-bi)+bi*A7)			
**8**	7	19.5	=IF(A8<cc,ao+ai*A8,ao+cc*(ai-bi)+bi*A8)		**SS_res_**	=SUMXMY2(B2:B21,C2:C21)
**9**	8	22.5	=IF(A9<cc,ao+ai*A9,ao+cc*(ai-bi)+bi*A9)			
**10**	9	24.3	=IF(A10<cc,ao+ai*A10,ao+cc*(ai-bi)+bi*A10)		**R^2^**	=1-(SUM((B2:B21-C2:C21)^2)/ SUM((B2:B21-ymean)^2))
**11**	10	24.8	=IF(A11<cc,ao+ai*A11,ao+cc*(ai-bi)+bi*A11)			
**12**	11	24.4	=IF(A12<cc,ao+ai*A12,ao+cc*(ai-bi)+bi*A12)			
**13**	12	23.9	=IF(A13<cc,ao+ai*A13,ao+cc*(ai-bi)+bi*A13)			
**14**	13	26.1	=IF(A14<cc,ao+ai*A14,ao+cc*(ai-bi)+bi*A14)			
**15**	14	28.4	=IF(A15<cc,ao+ai*A15,ao+cc*(ai-bi)+bi*A15)			
**16**	15	27.6	=IF(A16<cc,ao+ai*A16,ao+cc*(ai-bi)+bi*A16)			
**17**	16	25.0	=IF(A17<cc,ao+ai*A17,ao+cc*(ai-bi)+bi*A17)			
**18**	17	29.7	=IF(A18<cc,ao+ai*A18,ao+cc*(ai-bi)+bi*A18)			
**19**	18	27.3	=IF(A19<cc,ao+ai*A29,ao+cc*(ai-bi)+bi*A29)			
**20**	19	28.1	=IF(A20<cc,ao+ai*A20,ao+cc*(ai-bi)+bi*A20)			
**21**	20	30.2	=IF(A21<cc,ao+ai*A21,ao+cc*(ai-bi)+bi*A21)			

**Fig. 1:**
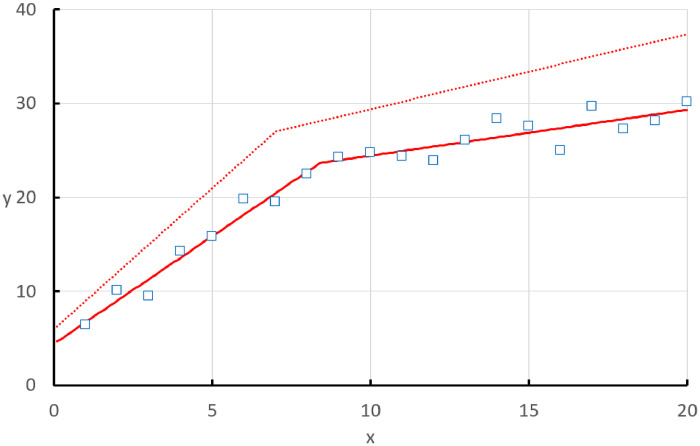
The observed data (open blue squares) with the fits according to the initial parameters estimates (offset red dotted line) and the best fit (bold red line) superimposed. A clear transition point is apparent from the best fit between 8 and 9.

2. The data are plotted as open blue squares on a scatter plot (Fig. 1), revealing an abrupt change in the data at about 8 on the x-axis, where the linear function describing the data decreases in slope.

3. The cells containing the parameters that determine the model are now addressed. Enter the labels a_o_, a_i_, b_i_ and cc in cells E2 to E5, respectively, then name cells F2 to F5 a_o_, a_i_, b_i_ and cc, respectively, using Name –> Define under the Insert menu.

4. Enter the following initial parameter estimates for a_o_, a_i_, b_i_ and cc as 6.0, 3.0, 0.8 and 7.0, respectively.

5. Label Column C as y_model_. The formula entered in Column C that defines the model is based on the following rearrangement of the functions. If the transition point occurs at cc on the x axis, then the data for x 〈 cc, referred to as the 1st function, is described by *y* = a_o_ + a_i_x, where a_o_ is the intercept with the y axis at *x* = 0, and a_i_ is the slope of the line. Where x 〉 cc, the linear function, referred to as the 2nd function, is *y* = b_o_ + b_i_x, with b_o_ as the intercept with the y axis at *x* = 0 and b_i_ is the slope of the line. However, we need to introduce a parameter for the transition point into the model. At the transition point (cc), which defines the intercept of the two lines:

$$a_{o}+a_{i}x=b_{o}+b_{i}x$$

$$b_{o}=a_{o}+\left(a_{i}-b_{i}\right)x$$

which equals b_o_ = a_o_ + cc * (a_i_ – a_o_) at the transition point.

Accordingly, where x 〈 cc, *y* = a_o_ + a_i_x; where x 〉 cc, *y* = a_o_ + cc * (a_i_ – b_i_) + b_i_x.

Enter the following formula in cell C2 then drag down to cell C21.

=IF(A2<cc,ao+ai*A2,ao+cc*(ai–bi)+bi*A2). The Boolean logic contained in this formula dictates that the 1st function is calculated when *x* < cc, but the 2nd function applies when *x* > cc.

6. Plot the data in Column C on the scatter plot, which will appear as the dotted red line offset from the data points (Fig. 1).

7. Label cells E6, E8 and E10 as y_mean_, SS_res_ and R^2^, respectively, then name cells F6, F8 and F10 as y_mean_, SS_res_ and R^2^.

8. In cell F6 enter the formula =AVERAGE(B2:B21), which calculates the mean of the data.

9. In cell F8 enter the formula =SUMXMY2(B2:B21,C2:C21). This specialised statistical function calculates the sum of the squares of the differences between the data and the model, known as the sum of the squares of the residuals (SS_res_).

10. In cell F10 enter the array formula =1–(SUM((B2:B21–C2:C21)^2)/SUM((B2:B21–ymean)^2)). This formula is equivalent to 1 – SS_res_/SS_total_.

11. The y_mean_, SS_res_ and R^2^ are computed as 21.89, 699.87 and 0.273, respectively.

*Application of SOLVER*

12. Open SOLVER under the Tools menu. In the ‘Set Objective’ box enter SS_res_. On the succeeding line check ‘min’. In the ‘By Changing Variables Cells’ box enter ao, ai, bi, cc. Press ‘Solve’ and the iteration process should yield a fit with parameter values for a_o_, a_i_, b_i_ and cc, of 4.45, 2.29, 0.49 and 8.41, respectively. The values of SS_res_ and R^2^ are calculated as 27.504 and 0.971 (Table 2). The best fit is illustrated as the bold red line in Fig. 1, with the transition point (cc) calculated as 8.41.

**Table 2:** The spreadsheet template displaying the best fit of the data according to the model quantified in Column C, as calculated by SOLVER. Note how the parameter values have changed from the initial estimates in A and the value of R^2^ approaches 1.

	A	B	C	D	E	F
**1**	**x**	**y_data_**	**y_model_**			
**2**	1	6.4	6.73		**a_o_**	4.45
**3**	2	10.1	9.02		**a_i_**	2.29
**4**	3	9.5	11.31		**b_i_**	0.49
**5**	4	14.3	13.59		**cc**	8.41
**6**	5	15.8	15.88		**y_mean_**	21.89
**7**	6	19.8	18.17			
**8**	7	19.5	20.45		**SS_res_**	27.504
**9**	8	22.5	22.74			
**10**	9	24.3	23.96		**R^2^**	0.971
**11**	10	24.8	24.45			
**12**	11	24.4	24.94			
**13**	12	23.9	25.43			
**14**	13	26.1	25.92			
**15**	14	28.4	26.41			
**16**	15	27.6	26.89			
**17**	16	25.0	27.38			
**18**	17	29.7	27.87			
**19**	18	27.3	28.36			
**20**	19	28.1	28.85			
**21**	20	30.2	29.34			

We expanded the method to incorporate non-linear functions, such that a combination of linear and non-linear functions can be deployed. For example, to fit data with an initial linear function followed by a power function, where the linear function is described by *y* = a_o_ + a_i_x and the power function is described by *y* =x^bi^ + b_o_, rearrange the equations in the manner described above to yield

$$=IF(A2<cc,ao+a*A2,ao+a*cc-cc^{b}i+A2^{b}i)$$

In a similar way fitting data with a linear function followed by a logarithmic function, where *y* = log_10_x, yields

=IF(A2<cc,ao+ai*A2,ao+(ai−bi)*LOG(cc,10)+bi*LOG(A2,10))

These expanded models can be incorporated into a spreadsheet template as described above.

The method is sufficiently flexible that any combination of functions can be used, and once the spreadsheet template has been established, allows for rapid throughput of data for analysis.

## Method validation

The method described in this paper allows users to carry out segmented regression analysis, a theoretically complex procedure, previously available only to expert mathematicians. The method uses the SOLVER add-in programme that is available in Microsoft Excel, which applies an iterative least squares algorithm to minimise the value of one cell by changing the values of other cells. Thus, in our method SOLVER minimises the sum of the squares between the observed data points and the model by optimising the parameters that determine the model. The method does not require advanced mathematical knowledge, but it does require the user to judge what is an appropriate model to fit their data.

There are numerous examples of biological systems that are suitable candidates for segmented regression analysis, where the model describes how a biological variable changes abruptly once a particular transition point is reached, rather than a smooth increase in y as x increases. These include the height/weight ratio of children as a function of age [14], the epinephrine increases in response to hypoglycaemia [15] and the serum level of cortisol in depressed patients [1].

We compared our method against historical data [1] and found an exact match between the estimated transition points. We also used the piecewise_func function in MATLAB as an additional validation step. which matched our method (see Supplementary Material).

Segmented regression continues to be a popular analytical method as indicated by these recent references [[16], [17], [18], [19]].

### Estimates of goodness of fit

It is desirable to quantify how well the model describes the data, as such measures of fit may suggest more appropriate better fitting alternative models. This is routinely assessed as the coefficient of determination (R^2^), which compares the variability of the data around the mean, with the variability of the data around the model [20]. The total variability (SS_total_) in the system is calculated as the sum of the squared differences between each data point and the mean: ∑(y_data_ – y_mean_)^2^. This is equivalent to variance in standard descriptive statistics. If there were no relationship between the dependent and independent variables, then we would expect a random variation about the mean. However, if the data are described by a model, then some of the variation is explained by the deviation of the model from the mean, called the explained error and quantified as the sum of the squares of regression (SS_reg_): ∑(y_model_ – y_mean_)^2^. However, most of the data points will not fall exactly on the model, so must be caused by other sources, called the unexplained error, which is the sum of the squared differences between each data point and the model and quantified as the sum of the squares of the residuals (SS_res_): ∑(y_model_ – y_data_)^2^. Since SS_total_ = SS_reg_ + SS_res_, R^2^ can be quantified according to two complementary relationships, either as the ratio of the SS_reg_/SS_total_ or as the complement of the ratio of SS_res_/SS_total_. This second relationship implies an alternative means by which SOLVER can optimize the fit of the model to the data by maximising the value of R^2^. As R^2^ approaches 1 the greater the total variance is explained by variance around the mean with a minimal contribution from the SS_res_ i.e. the data points are very similar to the fit.

## Limitations

The greater the number of parameters in the funciton the longer SOLVER will take. SOLVER requires fairly accurate initial estimates of the parameter values, otherwise the iteration process may proceed in the wrong direction and a solution is not found. Best practice is to use the graphically displayed initial fit as a visual guide for optimising parameter estimates prior to implementing SOLVER. The method we describe is limited to two functions and relatively simple data i.e. few data points. As data becomes more complex the user may need to customize the fitting protocol with additional constraints or increasing tolerance, which will slow the time taken for SOLVER to reach a solution, thus the method we describe is best suited for simple data.

## Ethics statements

Not applicable.

## Supplementary material *and/or* additional information [OPTIONAL]

Not Applicable.

## CRediT authorship contribution statement

**Amy J Hopper:** Data curation, Formal analysis, Writing – review & editing. **Angus M Brown:** Conceptualization, Methodology, Writing – original draft, Writing – review & editing.

## Declaration of competing interest

The authors declare that they have no known competing financial interests or personal relationships that could have appeared to influence the work reported in this paper.

## Data Availability

No data was used for the research described in the article.
